# Oxidative Stress, Tumor Microenvironment, and Metabolic Reprogramming: A Diabolic Liaison

**DOI:** 10.1155/2012/762825

**Published:** 2012-05-13

**Authors:** Tania Fiaschi, Paola Chiarugi

**Affiliations:** Department of Biochemical Sciences, University of Florence, 50134 Florence, Italy

## Abstract

Conversely to normal cells, where deregulated oxidative stress drives the activation of death pathways, malignant cells exploit oxidative milieu for its advantage. Cancer cells are located in a very complex microenvironment together with stromal components that participate to enhance oxidative stress to promote tumor progression. Indeed, convincing experimental and clinical evidence underline the key role of oxidative stress in several tumor aspects thus affecting several characteristics of cancer cells. Oxidants influence the DNA mutational potential, intracellular signaling pathways controlling cell proliferation and survival and cell motility and invasiveness as well as control the reactivity of stromal components that is fundamental for cancer development and dissemination, inflammation, tissue repair, and *de novo* angiogenesis. This paper is focused on the role of oxidant species in the acquisition of two mandatory features for aggressive neoplastic cells, recently defined by Hanahan and Weinberg as new “hallmarks of cancer”: tumor microenvironment and metabolic reprogramming of cancer cells.

## 1. Introduction

**Figure 1:**
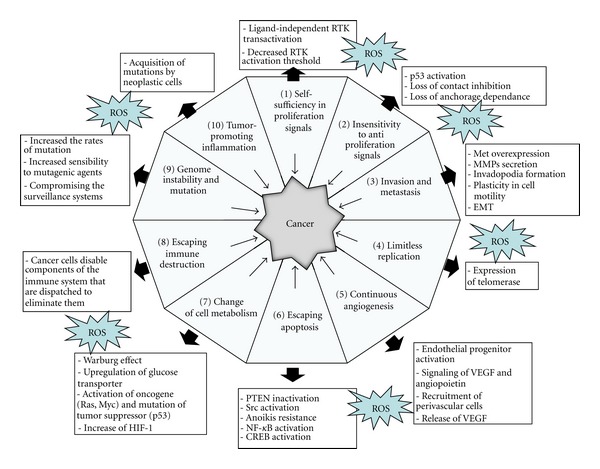
ROS play multiple roles in the hallmarks of cancers. Contribution of oxidants is indicated for each point (see text for details). (1) Self-sufficiency in proliferation signals: most normal cells wait for an external message before dividing. Conversely, cancer cells often counterfeit their individual proliferative messages. ROS a play role in ligand-independent RTK transactivation, decreased RTK activation threshold [11, 12]. (2) Insensitivity to antiproliferation signals: as the tumor enlarges, it squeezes adjacent tissues and therefore receives messages that would normally stop cell division. Malignant cells ignore these command. ROS are involved in p53 activation, loss of contact inhibition, and loss of anchorage dependence [12–14]. (3) Invasion and metastasis: cancers usually lead to death only after they overcome their confines to the particular organ in which they arose. Cancer cells need to escape the primary tumour, invade matrix of different organs, find a suitable metastatic niche, and then grow in this secondary site. ROS play a role in Met overexpression, matrix metalloproteinase secretion, invadopodia formation, and plasticity in cell motility, EMT [12, 15–18]. (4) Limitless replication: healthy cells can divide no more than 70 times, but malignant cells need more than 70 cycles to make tumours. Hence tumours need to enforce the reproductive limit of cells. ROS are involved in expression of telomerase [12, 19]. (5) Continuous angiogenesis: tumour is characterized by a chronically activated angiogenesis due to an unbalanced mix of pro-angiogenic signals thus sustaining cancer “feeding.” ROS play role in: endothelial progenitor activation, signalling of VEGF and angiopoietin, recruitment of perivascular cells, release of VEGF [20–22]. (6) Escaping apoptosis: in healthy cells, several conditions (including genetic damage or lack of ECM adhesion) activate a suicide program, but tumour cells bypass these mechanisms, thereby surviving to death messages. ROS are involved in PTEN inactivation, Src activation, Anoikis resistance, NF-*κ*B activation, and CREB activation [4, 12, 23–25]. (7) Change of cell metabolism tumours have the capability to modify or reprogram cellular metabolism to successfully carry on the neoplastic progression. ROS are involved in Warburg effect, upregulation of glucose transporter, activation of oncogene (Ras, Myc) and mutation of tumour suppressor (p53), and increase of HIF-1 [12, 26–28]. (8) Escaping immune destruction tumours acquire the capability to evade natural immunological destruction by T and B lymphocytes, macrophages, and natural killer cells [29]. Furthermore, there are two additional characteristics facilitating the acquisition of aggressive features called “enabling characteristics.” (9) Genome instability and mutation genomic alteration due to epigenetic mechanisms, the increase rate of mutation, or enhanced sensitivity to mutagenic agents can drive tumour progression. ROS are involved in increasing the rates of mutation, increasing sensibility to mutagenic agents, and compromising the surveillance systems [12, 29, 30]. (10) Tumor promoting inflammation innate immune cells, which are designed to fight infections and heal wounds, inadequately support the acquisition of hallmark capabilities with this leading to tumor expansion. ROS are involved in acquisition of mutation by neoplastic cells, thus accelerating their evolution towards heightened malignancy [29, 31].

With over 3 million novel cases each year in Europe, cancer is a main public health hitch with a vital need for new therapies. Hanahan and Weinberg defined in the 2000s the so-called hallmarks of cancers, mandatory characteristics of virtually all neoplastic cells, enabling them to grow in a foreign and hostile environment and allowing escaping endogenous protective systems [1]. These hallmarks are listed in our reinterpretation of the Hanahan and Weinberg picture (Figure 1). Firstly, we mention self-sufficiency in growth signals, that is, the ability of several cancer cells to produce in autocrine manner growth factors and cytokines, as well as the development of compensatory mechanisms enhancing growth factor receptor. activation [2, 3]. The insensitivity to natural growth arrest signals, as the abolishment of cell contact inhibition, and the ability to evade apoptosis are two other intimately correlated cues of neoplastic cells [2–4]. Cancer cells evade apoptotic death due to lack of cell adhesion, a process called *anoikis*, as well as death induced by several chemotherapeutic drugs, thereby leading to chemoresistance, at present the main obstacle to fight cancer dissemination [3–8]. Beside evasion from apoptotic death, cancer cells also escape senescence and the limiting in lifespan, overcoming immortalization. Last, they achieve two further features, which strongly facilitate dissemination of metastatic colonies and repopulation tumors elsewhere. Indeed the ability to recruit *de novo* formed vessels, the so-called neoangiogenesis or vasculogenesis, is mandatory first to grant nutrient supply once the tumor is grown and need new vasculature, and then to create a new way to reach the circulation and disseminate metastases to other organs [9]. Last, aggressive tumors increase their ability to invade surrounding tissues by enhancing their motility and ability to proteolytically degrade basal membrane and extracellular matrices [10]. 

Our paper is built upon mounting evidence that oxidative stress underlies many of the hallmarks of cancer as defined by Hanahan and Weinberg [29]. Studies in several cancers, including breast, prostate, and colon carcinoma, as well as melanoma, have clearly established that oxidative stress players are expressed aberrantly in cancers and positively affects mandatory steps of cancer initiation and progression, by acting on cell proliferation and anchorage independent cell growth, causing insensitivity to apoptosis, sustaining *de novo* angiogenesis, and by altering the migration/invasion programme through metabolic and epigenetic mechanisms (Figure 1). In this scenario, Reactive Oxygen Species (ROS) exert a key role affecting several hallmarks of cancer. Indeed, ROS are involved in proliferation by a ligand-independent transactivation of receptor tyrosine kinase and ERK activation as well as in promoting tissue invasion and metastatic dissemination due to metalloproteinase secretion/activation and epithelial mesenchymal transition. In addition, ROS are involved in tumor angiogenesis, through the release of vascular endothelial growth factor and angiopoietin and for evading apoptosis/*anoikis* [3, 4, 13, 20, 32–37].

In cancer cells, high levels of ROS can result from increased basal metabolic activity, mitochondrial dysfunction due to hypoxia or mitophagy, peroxisome activity, uncontrolled growth factor of cytokines signaling, and oncogene activity, as well as from enhanced activity of known ROS sources as NADPH oxidase (NOXes), cyclooxygenases (COXes), or lipoxygenases (LOXes) [11, 38, 39]. Compelling experimental and clinical evidence indicates that ROS can promote many aspects of tumour onset and progression towards a malignant phenotype. In general, the activity of oxidants on tumors can depends on (i) their mutagenic potential, a mandatory factor for tumor initiation [40], (ii) their effects on intracellular signaling pathways controlling cell proliferation and survival [32, 33]; (iii) their impact on cell motility and invasiveness [12, 40], and (iv) their recognized role in stromal reactivity, mandatory for cancer development and dissemination, like inflammation, tissue repair, and *de novo* angiogenesis [40, 41]. 

Consequences of the production of oxygen radicals on cancer biology are pleiotropic and complex. Currently, our incomplete knowledge of the entire network of reactions and effects profoundly hinders the implementation of novel and effective redox-based anticancer strategies. In fact, besides being directly involved in mutagenesis and genomic instability, ROS also contribute epigenetically to cancer development and progression, by acting as signalling intermediates downstream of mitogen receptors and adhesion molecules and as inducers of genetic programs leading to cell invasion and malignancy. Furthermore, oxidation of cell constituents is a general cause of cell stress and promotes spontaneous and therapy-induced tumor cell death by making cells more vulnerable. Resistance to oxidative stress appears to be a major mechanism of tumor chemo- and radioresistance. Such diverse biological effects likely reflect distinct biochemical mechanisms operating in different compartments within cells. 

Recently, both tumor microenvironment and metabolic reprogramming have been included in the Hallmarks of Cancer model, in a revised perspective of the old hallmarks [29]. Both features, enabling cancer cells to achieve a more aggressive phenotype, have been correlated to oxidative stress and will be described below.

## 2. Tumor Microenvironment and Oxidative Stress

**Figure 2:**
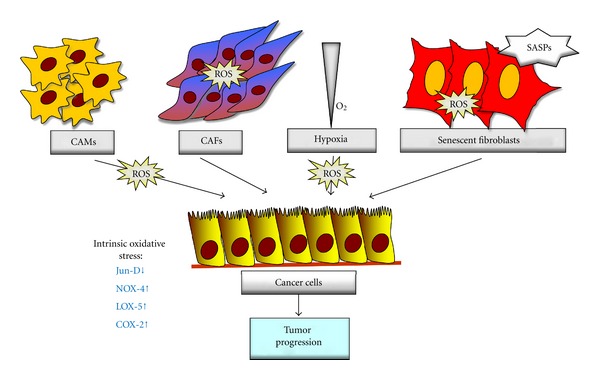
Oxidative stress in tumor microenvironment. Within microenvironment, oxidative stress can have intrinsic or extrinsic origin. Some stromal components can directly produce ROS. CAMs generate ROS through NOX2 activation and RNS through iNOS, while hypoxia produces oxidant species by deregulation of the complex III of mitochondrial electron transport or by NADPH oxidase activity [16, 42–46]. In response to extrinsic or intrinsic oxidative stress, CAFs became activated thus producing cytokines and proteases that affect tumour progression [41, 47, 48]. In addition, microenvironment or ageing-induced oxidative stress leads to secretion of “Senescent Activated Secretory Pathway” (SASP) by senescent fibroblasts affecting both stroma and cancer cells to promote cancer progression [49, 50]. Finally, cancer cells exacerbate oxidant environment by intrinsic production of oxidative stress through down-regulation of Jun D or enhanced of NOX-4, LOX-5 and COX-2 activity [41, 48, 51–53].

Beside cell-autonomous process involving genetically transformed cancer cells exposed to intrinsic oxidative stress, the importance of stromal cell types populating the tumoral microenvironment is now well established. Indeed, tumor microenvironment may affect evolution of cancers towards aggressiveness and metastatic dissemination through both structure-and function- based (matrix composition, hypoxia, acidity) or cell-based (cancer associated fibroblasts (CAFs) or macrophages (CAMs), endothelial precursors, etc.) mechanisms. Several factors, such as hypoxia or presence of CAFs or CAMs, have already been proven to elicit a prooxidant environment deeply affecting tumor progression and metastasis spread in several cancer models [2, 40–42] (Figure 2). 

CAFs, originated either by resident fibroblasts or by recruitment of circulating mesenchymal stem cells [54, 55], become activated, in response to tumor-delivered factors, through a mesenchymal-mesenchymal transition (MMT) converting them into “activated fibroblasts” similarly to myofibroblasts [54, 56]. Fibroblasts activation is profoundly affected by oxidative stress in both neoplastic and fibrotic diseases [41, 47, 57, 58]. Oxidative stress in tumours can be either intrinsic or extrinsic. Indeed, in skin carcinogenesis model, TGF*β*1 increases the intracellular ROS level in stromal fibroblasts, which initiated the MMT and concomitant changes of gene expression, leading to the secretion of Hepatocyte Growth Factor, Interleukine-6, and Vascular Endothelial Growth Factor that result in proinvasive signals for migration of tumour cells [47]. In addition, Toullec et al. reported a link between myofibroblasts accumulation and the oxidative stress in different pathophysiological conditions (JunD-deficient animals, HER-2 amplified breast adenocarcinoma) [41], highlighting again the importance of oxidative stress in CAFs reactivity. Furthermore, in the diseased prostate stroma, MMT depends by Tumor Necrosis Factor *β*1-generated oxidative stress through NOX4 activation that leads to downregulation of ROS-scavenging enzymes such as glutathione peroxidase 3, thioredoxin reductase 1 and the selenium transporter selenoprotein P plasma 1 [51]. Finally, senescence is another factor greatly affecting stromal oxidative stress. Indeed, DNA damage accumulation associated with ageing is involved in deregulation of ROS generation and decrease of antioxidant defences [59]. Indeed, senescent fibroblasts generate an inflammatory environment through the secretion of proinflammatory cytokines and proteases called senescence-activated secretory pathways, SASPs [60]. SASPs comprise soluble signalling factors, chemokines, insulin-like growth factor-1, secreted proteases, tissue-type plasminogen activators, the uPA receptor, and the plasminogen activator inhibitors, which concur to transform senescent fibroblasts into proinflammatory cells that promote tumor progression [49, 50, 60]. 

CAMs, that concur with CAFs to promote a prooxidant environment, have been recruited into several kinds of tumours, where they exert their effects by different mechanisms [42]. Firstly, the continuous generation of ROS, due to activation of macrophage NOX-2 and inducible Nitric Oxide Synthase, could directly promote invasion and metastasis, through CAFs recruitment or MMPs activation. Besides, CAMs secrete proinflammatory cytokines, which coordinate the inflammatory response in neighbouring stromal and cancer cells, leading to cancer cells dissemination [31, 61]. 

A decreased oxygen pressure (hypoxia) has been reported to be linked to an increase of intracellular/mitochondrial ROS that synergizes with other effects due to hypoxia to promote tumour progression [21, 43]. Mammalian cells respond to hypoxia by activating stress signal response, which triggers hypoxia-inducible factor (HIF-) 1 and -2 transcription helpful for adaptation and survival in the hostile milieu [62]. When cells become hypoxic, hydroxylation of the *α* subunit of HIF is prevented, resulting in stabilization of the protein and activation of its transcriptional activity. HIF*α* stabilization occurs through ROS production due to electron transport chain failure or NADPH oxidase [21, 63]. Indeed, pharmacologic and genetic data point to ubiquinone cycle of complex III as the source of ROS generation during hypoxia to stabilize HIF1*α* protein [44, 62, 64, 65]. Intratumoral hypoxia can produce several different effects on cancer cells, ranging from metabolic reprogramming towards a glycolytic phenotype, overexpression of ABC transporters, selection of mutated cells whose apoptotic process is deficient, or protection from apoptotic inducers. Indeed, hypoxic cancer cells are more invasive, resistant to apoptosis and ultimately to chemotherapy and radiation therapy [66, 67]. Moreover, mounting evidence indicates that hypoxic cancer cells undergo exposure to oxidative stress, thereby developing adaptive strategies to survive to the hostile milieu [12, 22]. Of note, hypoxic cells can enhance their antioxidant capacity and hypoxia can behave as a promoting factor for this behaviour, with a possible correlation with resistance to therapy [11, 68]. 

It is important to underline that the adaptive strategies are indeed the antioxidant responses and that an anti-oxidant phenotype may result in increased aggressiveness.

We also recently reported that aggressive melanoma cells respond to hypoxia engaging a motogen escaping strategy, based on redox stabilization of HIF-1 and activation of the Met protooncogene, allowing a proteolytic motility enhancing metastatic dissemination to lungs [69]. In keeping with the key role exerted by ROS in sensing the effects of hypoxia, Gao et al. reported that the antitumorigenic effect of antioxidants as N-acetyl cysteine and vitamin C in murine models of Myc-mediated tumorigenesis is indeed HIF-1-dependent [70].

Thus, the adaptations to surrounding stromal cells, together with the intrinsic metabolic reprogramming of cancer cells (see below) lead to profoundly altered ROS production and sustained oxidative stress in tumor tissue [32, 33]. As a consequence, oxidant-sensitive transcription factors like Hypoxia Inducible Factor-1 (HIF-1) or Nuclear Factor *κ*-B (NF-*κ*B) become active and play a mandatory role in eliciting a promigratory and proinflammatory response in cancer cells [71–73]. In addition, human prostate CAFs exert their propelling role for EMT in strict dependence on cycloxygenase-2 (COX-2), NF-*κ*B, and HIF-1, due to COX-2-mediated release of reactive oxygen species, which is mandatory for EMT, stemness, and dissemination of metastatic cells [48, 59]. These responses, similarly elicited by several components of tumor microenvironment, like cancer-associated fibroblasts, hypoxia, or acidity, embrace enhanced motility, survival to stressful environment, and reconfiguration of metabolism. The motile response is commonly recognised as Epithelial Mesenchymal Transition (EMT), an epigenetic transcriptional program leading cells to lose epithelial features and achieve mesenchymal-like motility [74–77]. EMT has been correlated with achievement of stem-cell like cues, as increase in the ratio of expression of CD44 and CD24, increase in CD133 expression, and enhancement of anchorage-independent growth and spheroid formation, as well as selection of tumor initiating cells able to disseminate metastases [78, 79]. Again, both EMT and stemness have been reported as redox-sensitive and to exploit prooxidant environment to drive metastatic dissemination and resistance to chemotherapies in several cancer models [48, 80]. 

Beside the role played by stromal cells, EMT can be also elicited by intratumoral hypoxia, acting in a biphasic manner. Hypoxia-induced migration include an early mitochondrial delivery of ROS, leading to activation of cell polarization and oriented migration; then, there is a second delayed phase, in which ROS act on HIF-1*α* stabilization and VEGF expression, which sustains active motility [81]. In addition, stromal elements of tumor microenvironment regulate EMT and stemness through strengthening of hypoxic stimuli. By this way, CAFs are able to mimic the hypoxic stimuli, experiencing HIF-1 expression due to their oxidative stress, but without the real need for oxygen deprivation [41]. Indeed, exposure to reactive stromal fibroblasts engages an HIF-1 and NF-*κ*B-mediated transcriptional response driving EMT, but that does not need hypoxia [48]. Of course it is likely that the appearance of intratumoral hypoxia should exacerbate this EMT programme, enhancing the motile response (Comito, unpublished results).

Oxidative stress during ovarian tumorigenesis has been recently correlated with a stress signature involving two miR-200 family members, miR141 and miR200a, already implicated in the control of EMT and stemness [82, 83]. In particular, the paper of Mateescu et al. demonstrates that high-grade human ovarian adenocarcinomas that accumulate miR-200a contain high level of ROS, which correlate with improved survival of patients in response to treatment and conclude that although oxidative stress promotes tumor growth, it also sensitizes tumor to treatment, which could account for the limited success of antioxidants in clinical trials [84].

## 3. Metabolic Reconfiguration of Tumors Undergoing Oxidative Stress

Besides and in synergy with their altered perception of the tumor microenvironment, cancer cells undergo profound changes in their own intrinsic metabolism. The tendency of cancer cells to undergo Warburg metabolic reprogramming, characterized by increased activity in aerobic glycolysis and by lipid metabolism deregulation, is widely acknowledged. Recently both hypoxia and CAFs have been recognised to synergize in metabolic reprogramming of cancer cells, both establishing a sort of “Cori cycle” between glycolytic and respiring cells [85]. Indeed, hypoxia and/or contact with CAFs leads cancer cells to upload lactate, produced by neighbouring hypoxic cells or CAFs, which feeds aerobic cancer cells through respiration and anabolic functions [86, 87]. 

The reconfiguration of metabolism through oxidative stress occurring during cancer formation affects the metabolic flux and network topology of pathways in central carbon metabolism. It has been recently demonstrated that oxidative stress leads to mitophagy and limitation of oxidative phosphorylation [88] and to cysteine oxidation and inactivation of the M2 isoform of pyruvate kinase, with the consequence to enhance the level of glycolytic intermediates that are reconverted to the pentose phosphate cycle. This diverted pathway gives a key advantage to cells experiencing oxidative stress, which can use NADPH produced by pentose phosphate cycle and scavenge ROS, rescuing survival conditions. This adaptation was attributable to accumulation of phosphoenolpyruvate, due to redox inhibition of pyruvate kinase. Phosphoenolpyruvate acts as feedback inhibitor of the glycolytic enzyme triosephosphate isomerase, which activates the pentose phosphate pathway, increasing antioxidative metabolism and preventing ROS accumulation. NADPH also compensates for the oxidative stress caused in cancer cells undergoing nucleotide/fatty acid synthesis [89, 90]. These metabolic changes have effect on the transcriptome, allowing adaptation to cope with high ROS level, upregulating anti-oxidant defence systems, and helping cancer cells to reconfigure metabolic activity towards ROS detoxification, finally enhancing the ability to survive in a prooxidant environment.

Moreover, a crucial role in the regulation of Warburg effect in cancer cells has been proposed for mitochondrial SIRT3, which belongs to NAD-dependent deacetylase family, already involved in tumour metabolism. Indeed, the genetic loss of SIRT3 leads cancer cells to metabolic reprogramming towards glycolysis. This shift is mediated by an increase in cellular ROS generation that amplifies HIF-1*α* stabilization and HIF-1-dependent gene expression, thereby driving the tumor phenotype [91]. In addition, SIRT3 has been proposed as tumour suppressor via its ability to suppress ROS and regulate HIF-1*α* thus inhibiting tumour growth [92].

## 4. Conclusions

The possibility to target cancer cell malignancy by intervention on both its metabolic reprogramming and its interplay with environmental factors is now attracting several scientists. Effects of intratumoral hypoxia and/or infiltrating CAFs should in principle be targeted by disrupting the Warburg metabolism in both cancer and stromal cells, as well as their reconfiguration towards the pentose phosphate pathway antioxidant strategy. Promising pharmacological approaches include drugs targeting the lactate shuttle, as well as inhibitors of glycolysis combined with inhibitors of autophagy, a compensatory mechanism for nutrient starved cancer cells. Before developing such strategies, it will be essential to deeply investigate all biochemical reactions producing ROS within cancer cells, as well as their exact targets and downstream effects.
